# [^18^F]FMCH PET/CT biomarkers and similarity analysis to refine the definition of oligometastatic prostate cancer

**DOI:** 10.1186/s13550-021-00858-8

**Published:** 2021-11-27

**Authors:** Martina Sollini, Francesco Bartoli, Lara Cavinato, Francesca Ieva, Alessandra Ragni, Andrea Marciano, Roberta Zanca, Luca Galli, Fabiola Paiar, Francesco Pasqualetti, Paola Anna Erba

**Affiliations:** 1 Department of Biomedical Sciences, Humanitas University, Via Rita Levi Montalcini 4, Pieve Emanuele, Milan, Italy; 2 IRCCS Humanitas Research Hospital, Rozzano, Milan, Italy; 3 Nuclear Medicine, Department of Translational Research and Advanced Technology in Medicine and Surgery University of Pisa, Pisa University Hospital, Via Roma 67, 56123 Pisa, Italy; 4 MOX – Modeling and Scientific Computing, Department of Mathematics, Politecnico di Milano, p.zza Leonardo da Vinci 32, 20133 Milan, Italy; 5 CADS – Center for Analysis, Decision and Society, Human Technopole, Milan, Italy; 6 Medical Oncology, Pisa University Hospital, Via Roma 67, 56123 Pisa, Italy; 7 Radiation Oncology, Pisa University Hospital, Via Roma 67, 56123 Pisa, Italy; 8 University Medical Center Groningen, Medical Imaging Center, University of Groningen, Groningen, The Netherlands; 9 Department of Translational Research and New Technology in Medicine and Surgery, University of Pisa, Via Savi 10, 56126 Pisa, Italy

**Keywords:** [^18^F]FMCH PET/CT, Epithelial-mesenchymal transition, Number of lesions, Oligometastatic PCa, Radiomics, Silhouette index, Similarity analysis, Biomarkers

## Abstract

**Background:**

The role of image-derived biomarkers in recurrent oligometastatic Prostate Cancer (PCa) is unexplored. This paper aimed to evaluate [^18^F]FMCH PET/CT radiomic analysis in patients with recurrent PCa after primary radical therapy. Specifically, we tested intra-patient lesions similarity in oligometastatic and plurimetastatic PCa, comparing the two most used definitions of oligometastatic disease.

**Methods:**

PCa patients eligible for [^18^F]FMCH PET/CT presenting biochemical failure after first-line curative treatments were invited to participate in this prospective observational trial. PET/CT images of 92 patients were visually and quantitatively analyzed. Each patient was classified as oligometastatic or plurimetastatic according to the total number of detected lesions (up to 3 and up to 5 or > 3 and > 5, respectively). Univariate and intra-patient lesions' similarity analysis were performed.

**Results:**

[^18^F]FMCH PET/CT identified 370 lesions, anatomically classified as regional lymph nodes and distant metastases. Thirty-eight and 54 patients were designed oligometastatic and plurimetastatic, respectively, using a 3-lesion threshold. The number of oligometastic scaled up to 60 patients (thus 32 plurimetastatic patients) with a 5-lesion threshold. Similarity analysis showed high lesions' heterogeneity. Grouping patients according to the number of metastases, patients with oligometastatic PCa defined with a 5-lesion threshold presented lesions heterogeneity comparable to plurimetastic patients. Lesions within patients having a limited tumor burden as defined by three lesions were characterized by less heterogeneity.

**Conclusions:**

We found a comparable heterogeneity between patients with up to five lesions and plurimetastic patients, while patients with up to three lesions were less heterogeneous than plurimetastatic patients, featuring different cells phenotypes in the two groups. Our results supported the use of a 3-lesion threshold to define oligometastatic PCa.

**Supplementary Information:**

The online version contains supplementary material available at 10.1186/s13550-021-00858-8.

## Introduction

^18^F-fluoro-methyl-choline ([^18^F]FMCH) or ^11^C-choline positron emission tomography/computed tomography (PET/CT) is a well-established imaging modality to detect recurrence in Prostate Cancer (PCa) patients with biochemical failure after definitive treatment. About 30% of patients radically treated will recur [1], and the early diagnosis of disease relapse is pivotal to guide treatment decision [2–4]. The extensive use of functional imaging resulted in the identification of a new clinical disease entity namely oligometastatic disease (i.e., limited tumor burden) [5, 6].The theory of oligometastatic disease dates back to 1995 [7]. The interest in oligometastatic disease relies on biological features and clinical evidences resulting in better outcomes compared to plurimetastatic disease [8]. Consequently, the paradigm of metastatic PCa has evolved and, nowadays, tumor burden is an indicator for treatment allocation [5, 9–13]. The concept of oligometastatic disease is evolving by moving from a definition purely based on the number of lesions and/or involved organs to the identification of key features capable to biologically describe oligometastatic (and multimetastatic) phenotype to assign a patient to local or systemic treatment [14]. Quantitative imaging analysis can provide a number of biomarkers to be used in this setting. Indeed, advances in quantitative methods for data analysis have widened the scope of what imaging as a technology can enable in terms of scientific discovery. During the latest years, imaging data analysis have progressively shifted from a qualitative-semantic descriptors to quantitative measurements extracted by high-throughput methods able to make medical data mineable, namely radiomics. Accordingly, analysis of surface or volume, traditionally described by texture, has evolved in a discipline (in between image analysis and statistical modeling) aiming at describing tumor lesion heterogeneity through a multitude of quantitative indices (i.e., radiomic features). Hence, it's possible to capture information about the relations between pixels or voxels of a region or a volume of interest, by applying post-processing formulas to medical images acquired within the routinely clinical workflow of patients. Radiomic features can be extracted from any type of images from pictures, to pathology images, to cross-sectional images, to molecular imaging, to hybrid imaging [15, 16]. Although molecular imaging is largely used to define tumor burden and successfully select oligometastatic patients eligible for local treatment [17, 18], the role of image-derived biomarkers in oligometastatic PCa remains to be determined.

The present work aimed to evaluate the feasibility of radiomic analysis of [^18^F]FMCH PET/CT in patients with recurrent PCa after primary radical therapy. The goal of this research is two-fold. First, we investigated radiomic features extracted from [^18^F]FMCH PET/CT images according to the site of recurrence and tumor burden. Second, we explored intra-patient lesions similarity to provide biologically insights into oligometastatic (and plurimetastatic) recurrent PCa. Eventually, this approach provides an integrated multidimensional definition of oligometastatic recurrent PCa comprising image-derived features, going beyond the mere concept of the tumor burden.

## Materials and methods

### Study design and patient selection

All PCa patients eligible for [^18^F]FMCH PET/CT presenting with biochemical failure after first-line curative treatments were invited to participate in this observational trial. Biochemical relapse was defined according to clinical guidelines [19]. This preliminary analysis consisted of 92 patients (mean age 72 ± 7 years, median age 72 years, range 55–85) with a positive [^18^F]FMCH PET/CT prospectively enrolled between January 2011 and February 2018 in the above-mentioned trial. Demographic and clinical data including age, Gleason score (GS) at diagnosis, prostate specific antigen (PSA) level at the time of [^18^F]FMCH PET/CT, primary treatment, and androgen deprivation therapy (ADT; if yes, ongoing or discontinued) were collected for all patients. Patients baseline characteristics are summarized in Table 1.

**Table 1:** Baseline characteristics

Variable	Mean (median)	Range (std)
Age	72.09 (71.68)	54.88–85.24 (7.03)
Total volume (mL)	16.41 (3.16)	0.22–207.70 (34.72)
Gleason score	7.37 (7.0)	5.0–9.0 (1.027)
PSA	18.16 (2.66)	0.09–591.0 (70.96)

Variable	Number (%)
Number of metastases	
Oligometastatic (< 3)	38 (41.30)
Plurimetastatic (≥ 3)	54 (58.70)
Oligometastatic (< 5)	60 (65.22)
Plurimetastatic (≥ 5)	32 (34.78)
Oligometastatic (3 ≤ *n* < 5)	22 (23.92)
Gleason category	
Gleason (≤ 7)	53 (63.10)
Gleason (> 7)	31 (36.90)
Ongoing therapy	
YES	33 (35.87)
NO	59 (64.13)
Initial therapy	
RP	23 (25.0)
RP + RT	52 (56.52)
RT	9 (9.78)

### PET/CT image acquisition and analysis

[^18^F]FMCH PET/CT were acquired at Regional Center of Nuclear Medicine of the Azienda Ospedaliero-Universitaria Pisana with an integrated PET/CT system General Electric Discovery 710 (General Electric Healthcare, Waukesha, WI, USA). Image acquisition was performed according to version 1.0 [20] and version 2.0 [21] of the European Association of Nuclear Medicine (EANM) guidelines until and from February 2015, respectively. Image acquisition protocol is detailed in Additional file.

PET/CT images were visually interpreted by two experienced nuclear medicine physicians (PAE and AM), aware of the patient's medical history. Lesions were anatomically designed as regional or distant metastases according to the TNM staging system [22]. Each patient was defined as oligometastatic or plurimetastatic based on [^18^F]FMCH PET/CT findings. We arbitrary decided to use the two most frequently applied definitions of oligometastatic PCa, and classify patients twice based on the number of lesions. Accordingly, oligometastic disease was defined as PCa patients having with up to three [23] and up to five lesions [24]. Lesions were semi-automatically segmented by the PET VCAR software (GE Healthcare, Waukesha, WI, USA) on a General Electric workstation. A volume of interest (VOI) was draw for each lesion and visualized on CT images to check the anatomical correspondence. Radiomic features (*n* = 42) were extracted from each VOI by using the LIFEx software [25] (http://www.lifexsoft.org). Image processing and calculation of image-derived features are reported in Additional file 1: Table S1, according to the IBSI reporting guidelines [26].

### Statistical analysis

Patient characteristics were summarized in frequency tables, and descriptive statistics was provided.

Univariate analysis was used to assess the discriminant power of radiomic features, according to pre-specified clinical categories belonging to patients (i.e., oligometastatic versus plurimetastatic, GS ≤ 7 versus GS > 7) and lesions description (regional lymph nodes versus distant lymph nodes, and regional or distant lymph nodes versus bone metastases). The Mann–Whitney test (two-tailed) was used to evaluate marginal significance of each radiomic variable in the considered population. To capture the potential impact of ongoing ADT on textural features, such analysis was performed considering i) all patients, ii) the subset of patients undergoing ADT therapy, and iii) the subset of patients not on ADT. Significance has been confirmed with *p* ≤ 0.01 for GS and *p* ≤ 0.001 for all the other clinical categories.

Furthermore, PSA was considered apart from radiomic variables. PSA distribution in different groups was compared slicing patients according to pre-specified clinical categories (i.e., oligometastatic versus plurimetastatic, GS ≤ 7 versus GS > 7). Tests were performed on PSA values , and patients belonging to different groups were compared in terms of PSA distribution. The entire population of patients and the patients undergoing or not ADT were considered separately. PSA significance has been confirmed with *p* ≤  00.05, and results were discussed accordingly. Analyses were carried-out on SPSS version 25.

Additionally, a similarity analysis aimed at explored intra-patient lesions heterogeneity was performed as detailed in Additional file 1. Intra-patient lesions' similarity was assessed using the silhouette index (i.e., similarity index) [27]. The silhouette index ranges from -1 to 1. A value close to 1 entails a high intra-patient lesions' similarity. Conversely, a negative silhouette index indicates a low intra-patient lesions' similarity, with proximity or even overlap of lesions belonging to different patients in the latent space. For all those patients with just one lesion, the silhouette is set to zero and taken apart from any analysis. Similarity analysis included patient-based analysis, anatomy-based analysis, and metabolism-based analysis.

Kruskal test was used to compare intra-patient all-lesions' similarity in different groups obtained splitting patients according to pre-specified clinical categories (i.e., oligometastatic/plurimetastatic, Gleason Score ≤ 7/ > 7, median PSA in the population at the time of PET/CT > 1.93 ng/mL***/*** ≤ 1.93 ng/mL, and ongoing ADT treatment yes/no).

Unsupervised clustering was used as final step to support intra-patient lesions' similarity analysis interpretation. Radiomic-based clusters characterization was explored in order to check if any prevalence of the categories previously described could be appreciated. Analyses were carried-out on Python.

## Results

**Fig. 1:**
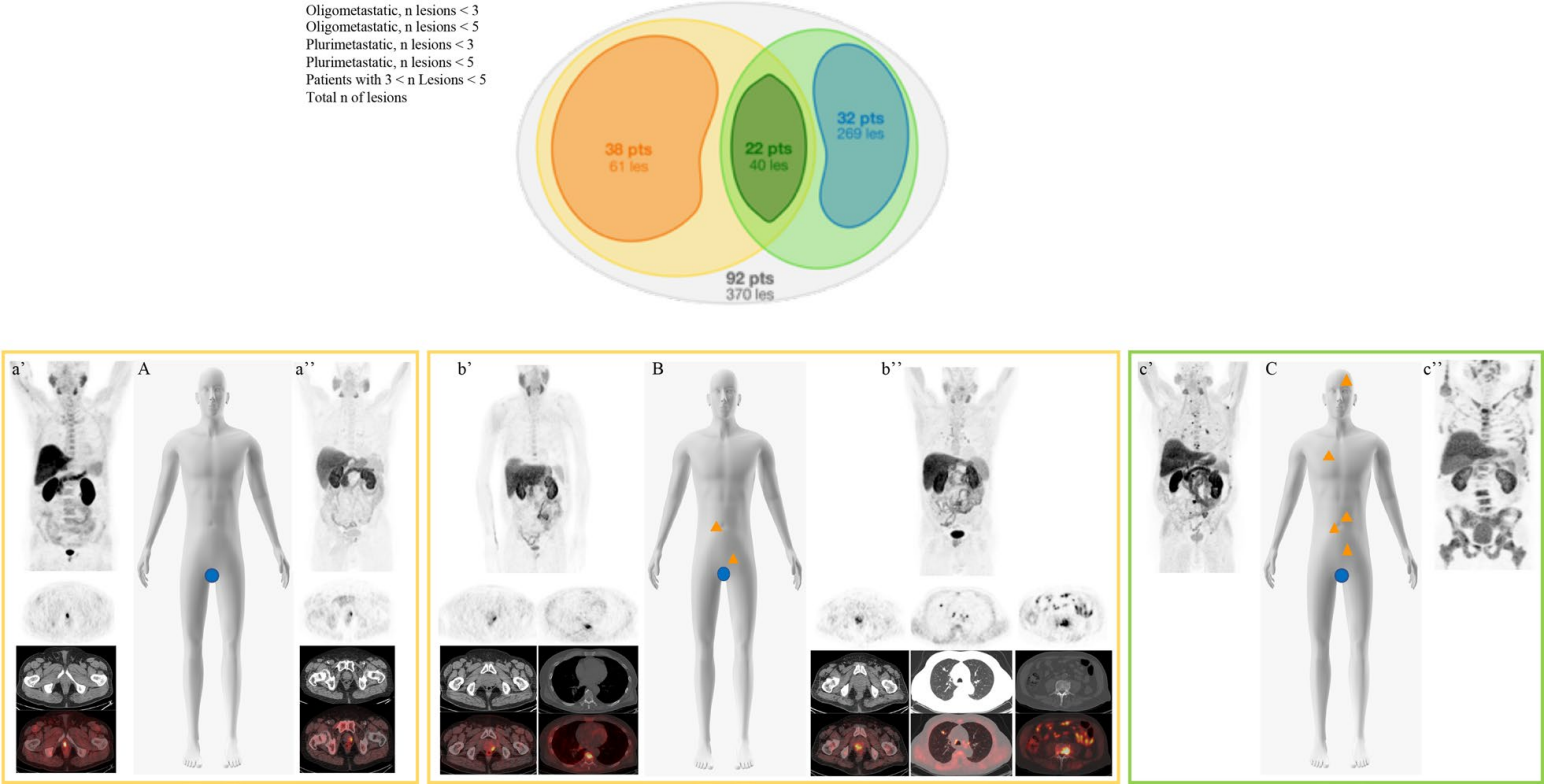
Schematic representation of the final classification in oligometastatic and plurimetastatic patients based on the number of lesions identified at [^18^F]FMCH (up to 3/5 and > 3/5, respectively) PET/CT findings at analysed population (upper panel). At lower panel clinical examples from the series: schematic representation (**A**–**C**) and [^18^F]FMCH PET/CT findings (**a**’–**a**’’, **b**’–**b**’’, **c**’–**c**’’, upper panel MIP images and lower panel top-down transaxial emission, CT and superimposed PET/CT at different levels) of oligorecurrent disease (**A**, **a**’, **a**’’), oligometastatic patients (**B**, **b**’, **b**’’) and multimetastatic patient (**C**, **c**’ and **c**’’)

[^18^F]FMCH PET/CT identified 370 lesions, anatomically classified as regional lymph nodes (*n* = 68) and distant metastases (*n* = 302). Distant metastases included distant lymph nodes (*n* = 81) and bone lesions (*n* = 221) (Additional file 1: Table S2). Using a cut-off of three lesions to define a limited tumor burden, 38 patients were oligometastatic and 54 plurimetastatic (Fig. 1). The number of oligometastic scaled up to 60 patients (thus 32 plurimetastatic patients) with a 5 lesions threshold (Fig. 1).

### Exploratory analysis

A number of features emerged as significant at univariate analysis (Additional file 1: Table S3) when considering tumor burden, GS, and distant or regional lymph nodes versus bone metastases. A significant trend toward a progressive reduction of the number of features was present moving form whole population, to the subset of patients under ADT to patients ADT-off.

Patients with plurimetastatic disease exhibited higher PSA values than patients with limited disease burden (Table 2), irrespectively of the criterion used for defining oligometastatic disease (up to 3 or 5 lesions), with a higher correlation using the 5 lesions threshold. PSA values also differed significantly between plurimetastatic disease with more than 5 lesions and patients with more than 3 and not more than 5 lesions (*p*-value = 0.001). In addition, PSA values were higher in patients with bone recurrence as compared to the ones with regional lymph nodes metastasis (*p*-value < 0.0001; Table 3). No difference in PSA levels were found in patients with a GS ≤ 7 or > 7 (*p* = 1) or in patients ADT-on or ATD-off (*p* = 0.07).

**Table 2:** Values of PSA at the time of PET/CT based on tumor burden

PSA	Oligometastatic ≤ 3	Multimetastatic > 3	*p*-value	Oligometastatic ≤ 5	Multimetastatic > 5	*p*-value
Mean	3.6959574	38.162941	0.0015	3.524	59.99095	< .0001
Dev std	5.3415206	107.03347		4.939003	132.6206	
Min	0.09	0.24		0.09	1.93	
Max	32	591		32	591	
Median	1.85	4.73		1.8	13.05

**Table 3:** Values of PSA at the time of PET/CT based on site of recurrence

PSA	Skeleton	Regional Ln	Distant Ln	*p*-value S—R	*p*-value S—D	*p*-value R—D
Mean	67.076039	9.3731667	18.236806	< 0.001	0.2291	0.0265
Dev std	148.03796	23.508696	43.819157			
Min	0.09	0.1	1.06			
Max	591	128	211			
Median	5.77	2.72	3.81			

### Similarity analysis

**Fig. 2:**
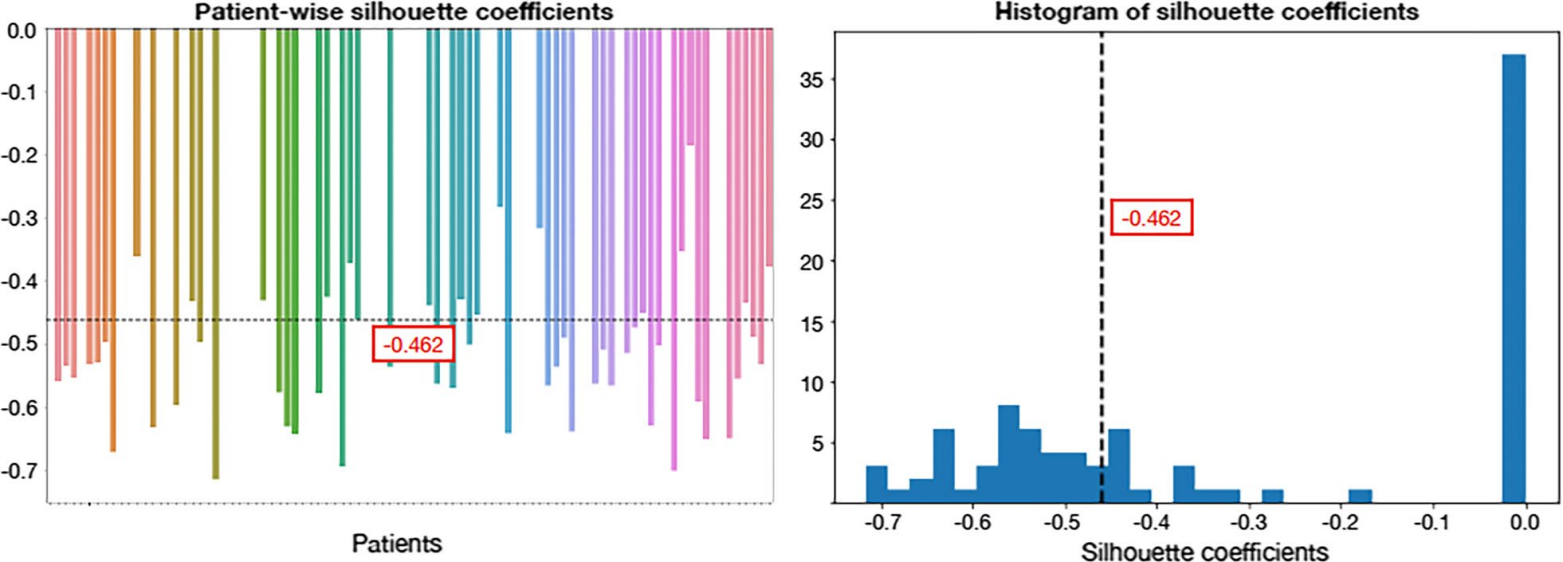
Lesions' similarity within each patient. Patients were included in the plot with no specific sorting criterium. Subjects were indeed randomly attributed a number from 1 to 92 as to be univocally identified. This number was used as agnostic coding factor. Colors are also randomly attributed to patients according to a palette of our choice, such that each subject could be associated to both a number and a color for visualization purposes

[^18^F]FMCH PET/CT identified multi-lesions disease in 55 patients. Patient-based analysis results are shown in Fig. 2. The mean similarity index was −0.462, indicating that lesions were poorly matched to the ones belonging to the same patient, and much more closed to other patients' lesions. Anatomy-based analysis results are shown in Additional file 1: Fig. S1. The mean similarity index was negative, regardless of the site of recurrence (−0.291 for regional lymph nodes, −0.343 for distant lymph nodes, and −0.429 for bone metastases). At the paired *t*-test, the mean anatomy-based similarity index was significantly different from the patient-based similarity index in all three cases (*p* < 0.01). Organ-specific lesions lead to a reduced intra-patient heterogeneity with respect to all the lesions. The mean similarity index of the metabolism-based analysis (Additional file 1: Fig. S2) was negative (−0.380 for the first tertile, −0.380 for the second tertile, and −0.428 for the third tertile), although showing lower heterogeneity when considering only the maximum standardized uptake value (SUV_max_)-wise homogeneous lesions for computing patients' silhouette. At the paired t-test significant differences between mean similarity index were found (*p* < 0.01), suggesting quite a concordance between higher radiomic features and SUV_max_ statistical parameter. Patients' lesions result into a more homogeneous clustering when sliced per SUV_max_ tertile while show higher heterogeneity when considered all together.

**Fig. 3:**
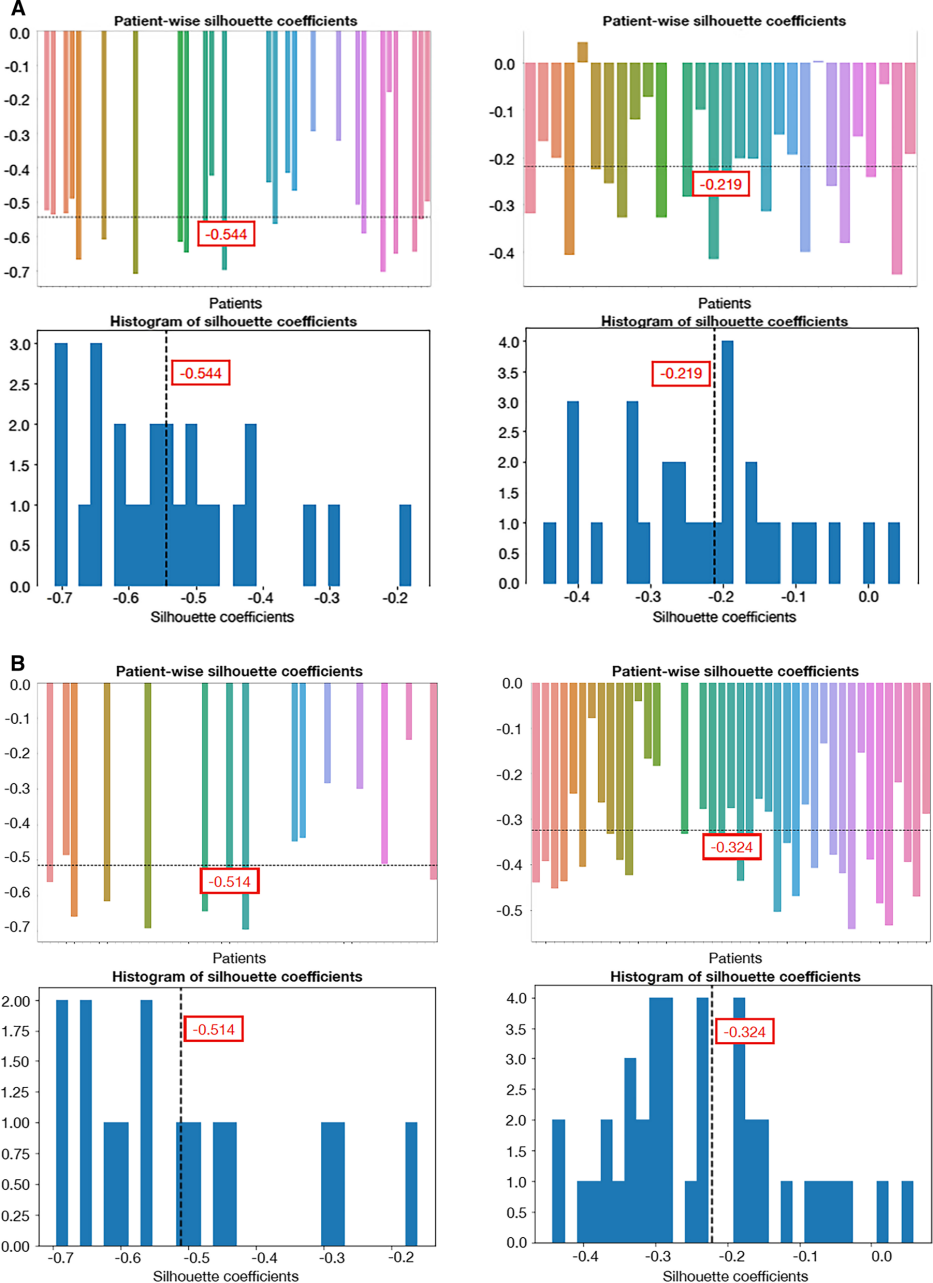
Intra-patient lesion's similarity according to tumor burden in multi- (left) and oligo-metastatic (right) patients, respectively. Oligometastic disease was defined as up to three lesions (**a**) or up to five (**b**)

Grouping patients according to the number of metastases, the 3-lesion threshold definition of oligometastatic exhibited lower heterogeneity than plurimetastic disease (Fig. 3a; *p* = 0.0003). The 5-lesions-threshold definition of oligometastatic disease showed a similar heterogeneity of plurimetastic disease (Fig. 3b; *p* = 1.337e−08). These findings were also confirmed in patients classified with the three lesions threshold as compared to patients with a lesion number between three and five and with more than five lesions (*p* = 7.223e−06). Patients with oligometastatic disease with a maximum of three lesions were less heterogeneous than the other two groups. Intra-patient lesion's similarity was comparable when considering patients with a GS ≤ 7 and > 7 (Additional file 1: Fig. S3; *p* = 0.4068), whereas low PSA patients exhibited lower yet not statistically different heterogeneity than patients with high PSA (Additional file 1: Fig. S4; *p* = 0.2723). ADT weakly affected intra-patient lesion's similarity (Additional file 1: Figure S5; *p* = 0.0057).

Unsupervised clustering identified two clusters as the best interpretable choice (Additional file 1: Figure S6). We found a higher prevalence of the SUV_max_ range 1 (i.e., first SUV_max_ tertile with respect to second and third tertile) in the second cluster, a higher prevalence of plurimetastatic disease (i.e., > 5 lesions) in the first cluster, and a higher prevalence of bone metastases disease with respect to nodal metastases in the first cluster.

## Discussion

PCa recurrence represents a biological complex setting including patients with oligorecurrence, oligometastatic single-organ or multi-organ disease as well as multi-organ plurimetastatic disease, this latter present in about 60% of our patients in this series. The setting of metastatic disease is still rather unexplored for radiomic analysis, and few data are available in literature. Among the open issues, we identified the lack of consensus on the number of lesions to be included in the radiomic analysis as well as the biological significance of different recurrent disease phenotypes i.e., multiple single-organ metastasis versus synchronous multi-organ metastasis. While radiomics in the setting of oligorecurrent disease is rather intuitive taking advantage from the workflow developed for primary tumor lesion, the approach to oligo-/plurimetastatic disease with a *per lesion* analysis is a pure simplistic model. Indeed, the presence of multiple metastasis in the same organ or the spread within different organs and/or parenchyma requires proper modeling in light of the biological complexity of each metastases. Consequently, an insightful patients' data transformation from "long" to "wide" format accounting for the relationship among all lesions is necessary. In this work, we performed an intra-patient similarity analysis exploring the silhouette index to quantify and assess such relationship. By this approach, we demonstrated the ability of [^18^F]FMCH PET/CT radiomic analysis to differentiate disease recurrence site, predict the Gleason score, and tumor burden (oligometastatic versus plurimetastatic disease) in recurrent PCa patients. Specifically, our data showed the coexistence of multiple texture phenotypes within one single organ or in the same patient as demonstrated by the high lesions heterogeneity. Such heterogeneity significantly decrease when considering only lesions within the same organ rather than all the lesions globally, and when focusing on metabolically similar lesions characterized by comparable SUV_max_ values.

Additionally, our analysis suggested that oligometastatic disease should be limited to patients with no more than five lesions, with further benefit of a more conservative criterion of a maximum of three lesion threshold. In our series, patients with plurimetastatic disease exhibited higher PSA values than patients with a limited tumor burden, with a higher correlation when the five lesions threshold was used. This evidence was further supported by the presence of statistically different PSA values in the groups of patients with more than five lesions and patients with more than three-five lesions. Moreover, intra-patient lesions' similarity showed a relationship between PSA levels and intra-patient lesions' heterogeneity (the lower the PSA, the lower the intra-patient lesions' heterogeneity). In addition, the similarity analysis showed that patients with oligometastatic PCa defined with a 5-lesion threshold presented lesions heterogeneity comparable to plurimetastic patients. Differently, lesions within patients having a limited tumor burden as defined by three lesions were characterized by less heterogeneity, thus featuring different cells phenotypes. Indeed, the key metabolic nodes associated with tumor heterogeneity are the biological prerequisite for a different [^18^F]FMCH uptake, as result of enhanced entry of long-chain fatty acids into the mitochondria or as precursors of eicosanoid metabolism, providing to oligometastatic and plurimetastatic disease (from low to high), either a highly proliferative phenotype or invasive epithelial-mesenchymal-transition-like phenotypes, respectively, as shown by in vitro experiments [28–31]. Therefore, the similarity index provides a tool for a better insight of oligometastatic PCa, going beyond [^18^F]FMCH PET/CT visual image analysis numbering the lesions [8]. This is a fundamental step in the debate around the proper definition of oligometastatic PCa in the attempt of integrate image-derived phenotype information of the "biological oligometastatic concept".

We have to acknowledge some limitations. Firstly, the sample size was relatively small. However, all images were prospectively obtained and processed using a standardized acquisition protocol and the same radiomic workflow, making results consistent. Secondly, it should be bear in mind that the concept of oligometastatic (and plurimetastatic) disease may be affected by the imaging modality (including the tracer) and its spatial resolution as well as by the clinical setting (i.e., diagnosis, recurrence, or relapse), making any definition an approximation. On the other hand, the use of the gold standard to properly classify oligometastatic patients is unfeasible in daily practice. Therefore, although each definition—either based on clinical or imaging data—is burdened by the inherent limitation(s) of the used method, its refinement trough new insights results in a patient management improvement toward precision medicine. Moreover, the innate limit of imaging is the detection of macroscopic disease, and despite the understandable advantage of microscopic disease detection (e.g., circulating tumor DNA), the role of imaging is still crucial. Oligometastatic disease is almost a continuum of risk and could seem inappropriate to express a so complex biological entity with a number. However, as above-mentioned the most used approaches to define oligometastatic PCa rely mainly on numbers. Our study, evaluating lesions heterogeneity, suggested an evidence-based threshold for oligometastatic/plurimetastatic PCa.

Thirdly, the evaluation of heterogeneity of relatively small lesions—as are typically those of biochemical recurrent PCa—may seems useless. However, we equally manage "oligometastatic" patients with one regional nodal recurrence and with bone/visceral metastases, even if they are probably affected by biologically different diseases. This analysis demonstrated that image-derived biomarkers effectively entail more information that those "captured" by visual analysis, and that they may be used to "in-vivo" study some biological behaviors of the disease. Fourthly, radiomic features were extracted from [^18^F]FMCH which works quite well if PSA is greater than 2 ng/mL, but which is known roughly half as sensitive as prostate-specific membrane antigen (PSMA). Therefore, although imaging shows early disease that is expected to become widespread, it cannot be distinguished from the biological significance of oligometastases [8]. Accordingly, we will expect that an even more restrictive threshold should be used to numerically namely oligometastatic PCa when using PSMA-based imaging.

Lastly, we evaluated tumor heterogeneity and intra-patients lesions' similarity without any correlation with patients outcome. However, it was out of the scope of the present work. Indeed, we make use of the radiomic features with the aim of quantitively identifying different PCa phenotypes within patients. We thus intend to provide a different perspective—and possibly a new vision—for imaging data exploitation for drawing insightful conclusion, under the "umbrella" of radiomics. Among the extensive, yet often inconclusive and methodologically lacking, literature that is emerging around radiomics-based machine learning models (i.e., machine learning methods applied to radiomic features extracted from images as is), we propose a sound statistical method for characterizing within-patient tumor heterogeneity. Quality of methodology is an essential requirement for inclusion of new evidence emerged by from trials [32], especially in the developing field of advanced image analysis [33, 34]. Appropriateness of clinical definitions and strength of radiomic workflow (including data analysis) should be established before to assess any prognostic or predictive role of image-derived parameters, to be sure they will be meaningful.

## Conclusions

[^18^F]FMCH PET/CT radiomic analyses provided invaluable information about tumor heterogeneity of PCa recurrence, entailing discriminant ability in differentiating the disease according to the site of recurrence and the tumor burden. Based on our model, the definition of oligometastatic PCa should include patients with no more than three lesions. Indeed, oligometastatic patients defined as having up to five lesions, exhibited a heterogeneity comparable to plurimetastic patients. Conversely, when the limited tumor burden was defined as more than three lesions, oligometastatic patients were less heterogeneous than plurimetastatic patients, featuring different cells phenotypes in the two groups. Such tumor heterogeneity has key metabolic nodes associated with high tumor [^18^F]FMCH uptake, highlighting the benefit of potential subpopulation-specific targets with important therapeutic implications by radiomics analysis.

## Supplementary Information

**Additional file 1**. Supplementary materials include a detailed description of image processing and calculation of image-derived features, methods used for similarity analysis, and results of visual analysis, univariate analysis and similarity analysis in specific groups of patients.

## Data Availability

The manuscript represents valid work and neither this manuscript nor one with substantially similar content under the same authorship has been published or is being considered for publication elsewhere. Paola Anna Erba had full access to all the data in the study and takes responsibility for the integrity of the data and the accuracy of the data analysis. Raw data are available on specific request to the corresponding author.

## Abbreviations

[^18^F]FMCH: ^18^F-fluoro-methyl-choline
ADT: Androgen deprivation therapy
EANM: European Association of Nuclear Medicine
GS: Gleason score
SUV_max_: Maximum standardized uptake value
PCa: Prostate cancer
PET/CT: Positron emission tomography/computed tomography
PSA: Prostate specific antigen
PSMA: Prostate-specific membrane antigen
VOI: Volume of interest
