# Supplementary material for: [18F]FMCH PET/CT biomarkers and similarity analysis to refine the definition of oligometastatic prostate cancer
Source: EJNMMI Res. 2021 Nov 27;11:119. doi: 10.1186/s13550-021-00858-8 (PMC8627538; doi:10.1186/s13550-021-00858-8)
Supplement: Supplementary file 1 — Additional file 1. Supplementary materials include a detailed description of image processing and calculation of image-derived features, methods used for similarity analysis, and results of visual analysis, univariate analysis and similarity analysis in specific groups of patients. [file 13550_2021_858_MOESM1_ESM.docx]

**Supplementary material**

**[^18^F]FMCH PET/CT radiomic and similarity analysis: the debate on the definition of oligometastatic prostate cancer could be over**

Sollini Martina^1,2^, Francesco Bartoli^3^, LaraCavinato^4^, Francesca Ieva ^4,5^, Alessandra Ragni ^4^, Andrea Marciano^3^, Roberta Zanca^3^, Luca Galli^5^, Fabiola Paiar^6^, Pasqualetti Francesco^6^ and Paola Anna Erba ^3,7^

^1^ Department of Biomedical Sciences, Humanitas University, Via Rita Levi Montalcini 4, Pieve Emanuele (Milan), Italy

^2^ IRCCS Humanitas Research Hospital, Rozzano (Milan), Italy

^3^ Nuclear Medicine, Department of Translational Research and Advanced Technology in Medicine University of Pisa and Pisa University Hospital, Via Roma 67, 56123, Italy

^4^ MOX – Modeling and Scientific Computing, Department of Mathematics, Politecnico di Milano, p.zza Leonardo da Vinci 32, 20133 Milano (Italy)

^5^ CADS – Center for Analysis, Decision and Society, Human Technopole, Milan (Italy)

^6^ Medical Oncology, Pisa University Hospital, Via Roma 67, 56123, Italy

^7^ Radiation Oncology, Pisa University Hospital, Via Roma 67, 56123, Italy

^8^ University of Groningen, University Medical Center Groningen, Medical Imaging Center, Groningen, The Netherland

Image acquisition protocol and processing

Image acquisition protocol and processing reported according to the IBSI reporting guidelines (25) are detailed in Supplementary Table 1. Briefly, dynamic PET/CT images (one minute per frame, for a total of 6 frames) started one minute after the intravenous injection of [^18^F]FMCH (of 4 MBq/kilogram of body weight) centred on the pelvis. Three-dimensional whole body images were acquired after a rest period of about 45 minutes. PET images were corrected for attenuation using the acquired unenhanced CT data (120 KV, 0.5 second per rotation, 3.27-mm reconstructed section thickness).

Both dynamic and static images were used for visual analysis, while radiomic features were extracted only from static ones.

| **Supplementary Table 1.** Image acquisition and processing parameters reported according to IBSI | |
| --- | --- |
| Imaging | PET |
| Region of interest | Whole body (from the skull base to the mid-thigh); standard protocol |
| Patient preparation | Fasting for at least 4 hours before injection. |
| Radioactive tracer | 18F-fluorocholine ([^18^F]FMCH); Intravenous administration  Injected activity range (4 MBq/kilogram of body weight) |
| Contrast agent | None |
| Acquisition and reconstruction & Scanners | General Electric Discovery 710  **(Static) PET CT Dynamic) PET CT**  **Min/bed position** 2.5 min x 6-8 (static) – 6 min x 1 (Dynamic) –  **Crystal** – LYSO  **Reconstruction** Iterative(VUE-PointFX 24subset 3 iterations),TOF Sharp IR–  **Procedure** width of the Gaussian filter (5mm) (FWHM) to spatially smooth intensities  **Attenuation correction** On CT data  **Matrix (pixels)** 256×256 512×512 256×256 512×512  **Resolution (mm)** 2.73×2.73 1.37×1.37 2.73×2.73 1.37×1.37  **Slice thickness (mm)** 3.27 3.27 3.27 3.27  **Slices** – 64 -- 64  **Voltage (kV)** – 120 – 120  **Tube current (mA) -** 140 – 140 |
| **Analysis** |  |
| Approach | The images were analyzed as a volume (3D). |
| Process structure | Image acquisition -> reconstruction -> anonymization -> export -> segmentation -> texture analysis -> feature calculation -> report |
| Software | Calculation of PET parameters was performed using the LIFEx software (24) (<http://www.lifexsoft.org>) |
| Data availability | All the original patient DICOM files are stored in the institutional PACS. Anonymized DICOM files and the results of the feature calculations are stored on the department hard disk. |
| **Data conversion** | |
| Procedure | No SUV normalization was applied to PET images. |
| **Segmentation** | |
| ROI | The volume of interest (VOI) included the tumour lesions. Textural features were calculated on PET images. |
| Procedure | PCa lesions were delineated on PET images applying a semi-automatic segmentation method, Lesions were semi-automatically segmented by the PET VCAR software (GE Healthcare, Waukesha, WI, USA) on a General Electric workstation. |
| **Resampling** |  |
| Voxel dimensions | 2*2*2 mm; PET: cubic interpolation |
| **Discretisation** |  |
| Discretisation method | PET: 64 bins (size bin equals to 0.3) between 0 and 20 SUV units, absolute resampling |
| **Feature calculation** |  |
| **PET Feature set** | Conventional/histogram-derived parameters **(HISTO)**  Skewness  Kurtosis  Entropy_Hist_  Energy_Hist_  Maximum  Mean  Standard deviation  Volume  TLA  **Shape and size**  Sphericity  Compacity  **Grey level co-occurrence (GLCM)**  Homogeneity  Energy_GLCM_  Contrast_GLCM_  Correlation  Entropy_GLCM_  Dissimilarity  **Neighbourhood grey level difference matrix (NGLDM)**  Contrast_NGTDM_  Coarseness  Busyness  **Grey level run length matrix (GLRLM)**  Short-run emphasis  Long-run emphasis  Low grey-level run emphasis  High grey-level run emphasis  Short-run low grey-level emphasis  Short-run high grey-level emphasis  Long-run low grey-level emphasis  Long-run high grey-level emphasis  Grey-level non-uniformity for run  Run length non-uniformity  Run percentage  **Grey level zone length matrix (GLZLM)**  Short-zone emphasis  Long-zone emphasis  Low grey-level zone emphasis  High grey-level zone emphasis  Short-zone low grey-level emphasis  Short-zone high grey-level emphasis  Long-zone low grey-level emphasis  Long-zone high grey-level emphasis  Grey-level non-uniformity for zone  Zone length non-uniformity  Zone percentage |

Methods for similarity analysis

Firstly, radiomic features were normalized to Z-score. Correlation-based criteria was used to exclude highly correlated features (>95%). Features not highly correlated were used in a Principal Component Analysis (PCA), and the resulting scores accounting for at least 95% of the total variability of the original data were preserved and used to evaluate intra-patient all-lesions’ similarity (patient-based analysis). In addition, we also performed organ-wise intra-patient lesions’ similarity (anatomy-based analysis) and SUV_max_-wise intra-patient lesions’ similarity (metabolism-based analysis). In the anatomy-base analysis, lesions of each patient were splitted according to the involved site (i.e. regional lymph nodes, distant lymph nodes, and bone), and PCA-based feature reduction was implemented as detailed above in order to assess organ-wise intra-patient similarity, resulting in three silhouette indexes per patient (one per organ). In the metabolism-base analysis, lesions within each patient were splitted according to their metabolism (pertaining to one of the SUV_max_ tertiles). Specifically, SUV_max_ distribution was observed and lesions were categorized into three groups as they fall into the first, second or third distribution tertile. SUV_max_ categories were thus used to evaluate similarity in uptake-wise homogeneous lesions. Again, PCA-based feature reduction was implemented as detailed above in order to assess SUV_max_-wise intra-patient similarity, resulting in three silhouette indexes per patient (one per tertile). Each silhouette value of the anatomy-based and the metabolism-base analyses was compared to the all-lesions silhouette of the patient-base analysis (intended as reference), by paired t-test.

| Supplementary Table 2s: results of visual analysis (GS = Gleason score) | | | | | | | | | | | |
| --- | --- | --- | --- | --- | --- | --- | --- | --- | --- | --- | --- |
| Lesions | Oligo ≤ 3 | Multi > 3 | Oligo ≤ 5 | Multi > 5 | GS 5 | GS 6 | GS 7 | GS 8 | GS 9 | GS category (<=7) | GS category (>7) |
| Skeleton | 16 | 193 | 51 | 170 | 39 | 0 | 77 | 39 | 37 | 118 | 74 |
| Ln locoregional | 23 | 45 | 35 | 33 | 0 | 3 | 40 | 7 | 11 | 43 | 18 |
| Ln distant | 16 | 65 | 30 | 51 | 9 | 0 | 47 | 8 | 14 | 56 | 22 |
| Total | 67 | 303 | 116 | 254 | 48 | 3 | 164 | 54 | 62 | 217 | 114 |

| Supplementary Table 3s: results of univariate analysis; grey cells (p≤0,001 and p≤0,01 for Gleason score) indicate high significance | | | | | | | | | | | | | | | | | | |
| --- | --- | --- | --- | --- | --- | --- | --- | --- | --- | --- | --- | --- | --- | --- | --- | --- | --- | --- |
|  | Oligo  vs  Multi (>3) | | | Oligo  vs  Multi (>5) | | | GS≤7  vs  GS>7 | | | Distant lymph nodes  vs  Bone metastases | | | Regional lymph nodes  vs  Distant lymph nodes | | | Regional lymph nodes  vs  Bone metastases | | |
|  | entire | ADT yes | ADT no | entire | ADT yes | ADT no | entire | ADT yes | ADT no | entire | ADT yes | ADT no | entire | ADT yes | ADT no | entire | ADT yes | ADT no |
| SUV mean | <0.001 | 0.001 | 0.065 | <0.001 | <0.001 | 0.009 | 0.070 | 0.021 | 0.326 | <0.001 | 0.135 | <0.001 | 0.033 | 0.277 | 0.788 | <0.001 | 0.046 | <0.001 |
| SUV std | <0.001 | <0.001 | 0.076 | <0.001 | <0.001 | 0.016 | 0.021 | 0.044 | 0.027 | 0.003 | 0.224 | 0.002 | 0.692 | 0.128 | 0.176 | 0.069 | 0.394 | 0.352 |
| SUV max | <0.001 | <0.001 | 0.073 | <0.001 | <0.001 | 0.009 | 0.063 | 0.059 | 0.116 | <0.001 | 0.020 | <0.001 | 0.474 | 0.490 | 0.505 | <0.001 | 0.382 | 0.001 |
| TLA ml | <0.001 | 0.001 | 0.156 | <0.001 | <0.001 | 0.011 | 0.296 | 0.401 | 0.544 | <0.001 | <0.001 | <0.001 | 0.054 | 0.082 | 0.897 | <0.001 | <0.001 | 0.000 |
| HISTO Skewness | 0.177 | 0.009 | 0.988 | 0.579 | 0.895 | 0.563 | 0.238 | 0.146 | 0.722 | 0.129 | 0.007 | 0.632 | <0.001 | <0.001 | 0.146 | 0.002 | 0.007 | 0.017 |
| HISTO Kurtosis | 0.774 | 0.294 | 0.386 | 0.524 | 0.883 | 0.550 | 0.669 | 0.181 | 0.497 | <0.001 | <0.001 | 0.045 | 0.028 | 0.013 | 0.437 | 0.185 | 0.817 | 0.288 |
| HISTO Entropy | <0.001 | <0.001 | 0.124 | <0.001 | <0.001 | 0.022 | 0.470 | 0.806 | 0.105 | <0.001 | <0.001 | <0.001 | 0.289 | 0.554 | 0.889 | <0.001 | 0.003 | 0.001 |
| HISTO Energy | <0.001 | <0.001 | 0.150 | <0.001 | <0.001 | 0.017 | 0.760 | 0.389 | 0.119 | <0.001 | 0.004 | <0.001 | 0.251 | 0.324 | 0.903 | <0.001 | 0.006 | 0.001 |
| SHAPE Volume ml | 0.014 | 0.024 | 0.682 | 0.002 | 0.008 | 0.201 | 0.068 | 0.092 | 0.371 | <0.001 | <0.001 | <0.001 | 0.631 | 0.167 | 0.463 | <0.001 | <0.001 | <0.001 |
| SHAPE Sphericity | 0.253 | 0.643 | 0.078 | 0.431 | 0.886 | 0.226 | 0.122 | 0.035 | 0.822 | <0.001 | <0.001 | 0.003 | 0.648 | 0.882 | 0.706 | 0.004 | 0.002 | 0.130 |
| SHAPE Compacity | 0.001 | 0.017 | 0.124 | <0.001 | 0.001 | 0.030 | 0.147 | 0.240 | 0.434 | <0.001 | <0.001 | <0.001 | 0.135 | 0.141 | 0.605 | <0.001 | <0.001 | <0.001 |
| GLCM Homogeneity | <0.001 | 0.009 | 0.044 | <0.001 | <0.001 | 0.012 | 0.063 | 0.082 | 0.137 | 0.613 | 0.084 | 0.060 | 0.626 | 0.502 | 0.459 | 0.253 | 0.115 | 0.300 |
| GLCM Energy | 0.002 | 0.005 | 0.350 | <0.001 | 0.001 | 0.045 | 0.139 | 0.096 | 0.639 | <0.001 | <0.001 | <0.001 | 0.692 | 0.161 | 0.294 | <0.001 | <0.001 | <0.001 |
| GLCM Contrast | <0.001 | 0.002 | 0.069 | <0.001 | <0.001 | 0.019 | 0.007 | 0.004 | 0.064 | 0.103 | 0.805 | 0.018 | 0.796 | 0.093 | 0.184 | 0.277 | 0.054 | 0.438 |
| GLCM Correlation | 0.235 | 0.171 | 0.933 | 0.062 | 0.061 | 0.559 | 0.547 | 0.158 | 0.670 | <0.001 | <0.001 | 0.006 | 0.090 | 0.960 | 0.074 | 0.035 | 0.067 | 0.500 |
| GLCM Entropy | 0.001 | 0.004 | 0.343 | <0.001 | <0.001 | 0.046 | 0.206 | 0.178 | 0.615 | <0.001 | <0.001 | <0.001 | 0.672 | 0.186 | 0.355 | <0.001 | <0.001 | <0.001 |
| GLCM Dissimilarity | <0.001 | 0.002 | 0.060 | <0.001 | <0.001 | 0.017 | 0.009 | 0.004 | 0.085 | 0.178 | 0.521 | 0.024 | 0.906 | 0.119 | 0.214 | 0.301 | 0.055 | 0.438 |
| GLRLM SRE | 0.002 | 0.047 | 0.040 | <0.001 | 0.002 | 0.031 | 0.046 | 0.089 | 0.072 | 0.001 | 0.001 | 0.137 | 0.102 | 0.540 | 0.228 | 0.552 | 0.008 | 0.832 |
| GLRLM LRE | 0.003 | 0.065 | 0.056 | <0.001 | 0.004 | 0.038 | 0.051 | 0.079 | 0.099 | 0.001 | <0.001 | 0.110 | 0.089 | 0.594 | 0.217 | 0.437 | 0.005 | 0.922 |
| GLRLM LGRE | <0.001 | 0.003 | 0.091 | <0.001 | <0.001 | 0.016 | 0.145 | 0.015 | 0.882 | <0.001 | 0.185 | <0.001 | 0.002 | 0.061 | 0.367 | <0.001 | 0.004 | <0.001 |
| GLRLM HGRE | <0.001 | <0.001 | 0.074 | <0.001 | <0.001 | 0.011 | 0.049 | 0.019 | 0.226 | <0.001 | 0.158 | <0.001 | 0.088 | 0.636 | 0.915 | <0.001 | 0.165 | <0.001 |
| GLRLM SRLGE | <0.001 | 0.003 | 0.090 | <0.001 | <0.001 | 0.016 | 0.149 | 0.016 | 0.887 | <0.001 | 0.172 | <0.001 | 0.002 | 0.055 | 0.387 | <0.001 | 0.003 | <0.001 |
| GLRLM SRHGE | <0.001 | <0.001 | 0.077 | <0.001 | <0.001 | 0.011 | 0.047 | 0.019 | 0.217 | <0.001 | 0.176 | <0.001 | 0.095 | 0.678 | 0.938 | <0.001 | 0.188 | <0.001 |
| GLRLM LRLGE | <0.001 | 0.004 | 0.081 | <0.001 | <0.001 | 0.014 | 0.113 | 0.012 | 0.765 | <0.001 | 0.293 | <0.001 | 0.002 | 0.061 | 0.301 | <0.001 | 0.009 | <0.001 |
| GLRLM LRHGE | <0.001 | <0.001 | 0.078 | <0.001 | <0.001 | 0.010 | 0.067 | 0.028 | 0.282 | <0.001 | 0.100 | <0.001 | 0.080 | 0.502 | 0.944 | <0.001 | 0.092 | <0.001 |
| GLRLM GLNU | 0.593 | 0.544 | 0.784 | 0.602 | 0.616 | 0.986 | 0.006 | 0.026 | 0.106 | <0.001 | <0.001 | <0.001 | 0.760 | 0.167 | 0.593 | <0.001 | <0.001 | <0.001 |
| GLRLM RLNU | 0.003 | 0.010 | 0.388 | <0.001 | 0.003 | 0.068 | 0.066 | 0.083 | 0.425 | <0.001 | <0.001 | <0.001 | 0.263 | 0.155 | 0.921 | <0.001 | <0.001 | <0.001 |
| GLRLM RP | 0.003 | 0.072 | 0.056 | <0.001 | 0.005 | 0.044 | 0.044 | 0.078 | 0.073 | <0.001 | <0.001 | 0.065 | 0.106 | 0.540 | 0.241 | 0.321 | 0.004 | 0.924 |
| NGLDM Coarseness | <0.001 | 0.017 | 0.103 | <0.001 | 0.001 | 0.008 | 0.230 | 0.797 | 0.193 | <0.001 | <0.001 | <0.001 | 0.813 | 0.553 | 0.327 | <0.001 | 0.058 | <0.001 |
| NGLDM Contrast | 0.004 | 0.023 | 0.374 | <0.001 | <0.001 | 0.303 | 0.003 | 0.001 | 0.109 | 0.381 | 0.036 | 0.715 | 0.669 | 0.540 | 0.331 | 0.966 | 0.306 | 0.595 |
| NGLDM Busyness | 0.005 | 0.030 | 0.200 | 0.002 | 0.002 | 0.229 | 0.012 | 0.122 | 0.014 | 0.044 | 0.067 | 0.167 | 0.031 | 0.752 | 0.139 | 0.974 | 0.210 | 0.624 |
| GLZLM SZE | 0.011 | 0.032 | 0.343 | <0.001 | <0.001 | 0.374 | 0.002 | 0.005 | 0.027 | 0.056 | 0.016 | 0.403 | 0.409 | 0.447 | 0.653 | 0.747 | 0.20 | 0.956 |
| GLZLM LZE | 0.028 | 0.162 | 0.195 | 0.008 | 0.025 | 0.166 | 0.019 | 0.061 | 0.054 | <0.001 | <0.001 | 0.028 | 0.349 | 0.343 | 0.452 | 0.022 | 0.001 | 0.217 |
| GLZLM LGZE | <0.001 | 0.003 | 0.116 | <0.001 | <0.001 | 0.022 | 0.141 | 0.016 | 0.877 | <0.001 | 0.173 | <0.001 | 0.005 | 0.066 | 0.452 | <0.001 | 0.006 | <0.001 |
| GLZLM HGZE | <0.001 | <0.001 | 0.090 | <0.001 | <0.001 | 0.015 | 0.046 | 0.021 | 0.207 | <0.001 | 0.176 | <0.001 | 0.138 | 0.782 | 0.865 | <0.001 | 0.201 | <0.001 |
| GLZLM SZLGE | <0.001 | 0.004 | 0.209 | <0.001 | <0.001 | 0.034 | 0.313 | 0.039 | 0.687 | <0.001 | 0.089 | <0.001 | 0.019 | 0.035 | 0.985 | <0.001 | 0.001 | <0.001 |
| GLZLM SZHGE | <0.001 | <0.001 | 0.133 | <0.001 | <0.001 | 0.033 | 0.018 | 0.009 | 0.100 | <0.001 | 0.325 | <0.001 | 0.190 | 0.984 | 0.828 | <0.001 | 0.544 | 0.001 |
| GLZLM LZLGE | 0.001 | 0.022 | 0.074 | <0.001 | <0.001 | 0.022 | 0.028 | 0.007 | 0.213 | 0.289 | 0.295 | 0.035 | 0.005 | 0.295 | 0.277 | <0.001 | 0.431 | 0.001 |
| GLZLM LZHGE | 0.001 | 0.013 | 0.268 | <0.001 | 0.001 | 0.043 | 0.279 | 0.849 | 0.260 | <0.001 | <0.001 | <0.001 | 0.075 | 0.061 | 0.593 | <0.001 | <0.001 | <0.001 |
| GLZLM GLNU | 0.049 | 0.090 | 0.728 | 0.010 | 0.082 | 0.122 | 0.006 | 0.029 | 0.071 | <0.001 | <0.001 | <0.001 | 0.017 | 0.020 | 0.198 | <0.001 | <0.001 | <0.001 |
| GLZLM ZLNU | <0.001 | <0.001 | 0.125 | <0.001 | <0.001 | 0.014 | 0.935 | 0.411 | 0.348 | <0.001 | <0.001 | <0.001 | 0.086 | 0.401 | 0.553 | <0.001 | <0.001 | <0.001 |
| GLZLM ZP | 0.006 | 0.056 | 0.176 | <0.001 | 0.001 | 0.130 | 0.003 | 0.008 | 0.036 | 0.001 | <0.001 | 0.081 | 0.448 | 0.121 | 0.517 | 0.130 | 0.001 | 0.547 |

Supplementary Figure 1s. Intra-organ lesion’s similarity within each patient with respect to (from left to right) skeleton lesions, regional lymph nodes and distant lymph nodes respectively.

Supplementary Figure 2s. Intra-burden lesion’s similarity within each patient with respect to (from left to right) SUVmax first, second and third tertile lesions respectively.

Supplementary Figure 3s. Intra-patient lesion’s similarity in patients with GS≤7 (left) and GS>7 (right) respectively.

Supplementary Figure 4s. Intra-patient lesion’s similarity in patients with PSA≤1.93 (left) and PSA>1.93 (right) respectively.

Supplementary Figure 5s. Intra-patient lesion’s similarity in patients with on-going ADT (left) and off-going ADT (right) respectively.

Supplementary Figure 6s. Unsupervised clustering interpretation according to explored variables: a) upper pieplots represent first cluster’s percentage of lesions’ number, site and SUV values; b) lower pieplots show second cluster’s percentage of lesions’ number, site and SUV values
